# Development of spatially variant photonic crystals to control light in the near-infrared spectrum

**DOI:** 10.1038/s41598-022-20252-1

**Published:** 2022-09-27

**Authors:** Andrew Volk, Amit Rai, Imad Agha, Tamara E. Payne, Jimmy E. Touma, Rudra Gnawali

**Affiliations:** 1 Applied Optimization, Inc., 3040 Presidential Dr. Suite 100, Fairborn, OH 45324 USA; 2 Department of Physics, University of Dayton, Dayton, OH 45469 USA; 3 Air Force Research Laboratory, Munitions Directorate, Eglin Air Force Base, FL 32542-6810 USA

**Keywords:** Optics and photonics, Applied optics, Optical materials and structures, Other photonics

## Abstract

Spatially Variant Photonic Crystals (SVPCs) have shown the ability to control the propagation and direction of light in the near-infrared spectrum. Using a novel approach for simplified modeling and fabrication techniques, we designed unique, spatially-varied, unit-cell structures to develop photonic crystals that maintain self-collimation and direction of light for desired beam tuning applications. The finite-difference time-domain technique is used to predict the self-collimation and beam-bending capabilities of our SVPCs. These SVPC designs and the simulation results are verified in laboratory testing. The experimental evidence shows that two-dimensional SVPCs can achieve self-collimation and direct light through sharp bends. The simplicity and quality of these designs show their potential for widespread implementation in modern devices. These SVPCs will serve as a unique solution to optical systems for optical computing, multiplexing, data transfer, and more.

## Introduction

As the need for advanced optical systems for computing, communication, and optical modulators continue to rise, so too does the need for improved optical components for precise, high speed, and high-quality control of light^1^. Since their discovery in the late 1980s^2,3^, the capabilities and applications of Photonic Crystals (PCs) have continued to grow and expand. While they come in many different forms, at their foundation, PCs are periodic nanostructures whose material and structural properties define photonic bandgaps that affect the motion of specific wavelengths of photons in the same way that semiconductors affect the motion of electrons^4^. Over the years, scientists have created a range of different geometric structures and design approaches for PCs in order to manipulate bandgaps so that the control of absorption and direction of light is possible for various wavelength regions and applications^5–8^. Each PC design has unique advantages and drawbacks for specific applications, types of fabrication, or wavebands of operation. One of these PC designs is a lattice structure composed of individual unit cells with carefully tailored fill fractions (*ff*), lattice constants, and sizes or scales^9^. The nano- and microscale geometries forming the uniform lattice allow unique optical properties to be developed such as tailored bandgaps that enable the structure to maintain the self-collimation of light as it propagates through the structure^5,10,11^.

The ability to maintain self-collimation through a uniform lattice is beneficial in the design of PCs for the control and direction of light, as manipulating the uniform lattice enables PCs to be applied to a range of applications without a dedicated waveguide structure. As long as the spacing used for self-collimation is maintained, the lattice structure can be manipulated in order to guide light in a desired direction^11,12^. Using this principle, we have developed Spatially Variant Photonic Crystals (SVPCs) capable of bending light around sharp bends and achieving multiplexing of dissimilar wavelengths of light in the Near-Infrared (NIR) spectrum through a single PC structure^13^; this is a novel achievement for optical systems and has potential benefits for improved efficiency and transmission in multiplexing and optical computing. Developing SVPCs operating in the NIR was previously a highly challenging endeavor due to fabrication and tolerance requirements. SVPCs for beam control in the NIR will open new avenues for imaging, data transfer, or communication. This system can provide higher data transfer speeds using optical instead of electrical signals as well as smaller scale systems when compared to mirror or waveguide systems and operating through free space as opposed to optical fibers. Although we have presented our approach for simulating two-dimensional (2D) SVPCs in previous publications^10,14^, we seek to explore three-dimensional (3D) simulations in this paper and present initial validation of these designs through fabrication and laboratory experiments. This work shows the benefit and potential of SVPCs for applications in the NIR spectrum using low-refractive index materials. We present our method and results of the design, simulation, fabrication, and characterization of unique 2D SVPC structures using the photopolymer IP-Dip, selected based on its low-refractive index, availability, and capabilities for nano- and microscale printing. Individual SVPCs as well as networks of these devices can improve the efficiency and manipulation of light over conventional optical systems for custom, small-scale devices.

## Results

### 2D and 3D SVPC designs

We apply spatial variation of unit cells to the PC lattice to develop SVPCs capable of maintaining self-collimation as light propagates through sharp bends. We show the PC’s control of light propagation by analyzing its bandgaps and Iso-Frequency Contours (IFCs) using the Finite-Difference Time-Domain (FDTD) method. IFCs are formed by the angular distribution of scattered photons in the far field, providing insight into the propagation direction of incident light. Light at similar frequencies to the IFCs passes perpendicularly through each face of the contour^15,16^. Light entering within the range of distributed k-vectors in the IFC remains collimated as it propagates through the structure. Uniform unit cells allow the structure to control light throughout the SVPC as it is directed through one IFC to the next, allowing us to define specific paths of light propagation within the SVPC. Keeping the angular variation between unit cells in the SVPC lattice within the adiabatic regime, implying that cascaded IFCs could be defined such that the angular rotation from one IFC to the next is well within the acceptance angle, ensures that light remains collimated as it is guided through the SVPC. The range of light capable of propagating through IFCs and, as a result, the SVPC structure is shown through the bandgap. The PC bandgap defines the wavelength region, or range of frequencies, that are blocked or transmitted through the structure^4,15^. We can tune this bandgap by manipulating the fill fraction (*ff*) and spatial variation of the PC to control the propagation of light. The *ff* is calculated from the ratio of substrates to holes in the PC structure, determined by the size and spacing of the unit cells of the lattice and maintained during spatial variation. While we choose to set the spacing and size of holes to determine the *ff*, one can also set the *ff* to determine values for spacing or the size of holes in their own PCs.

**Figure 1:**
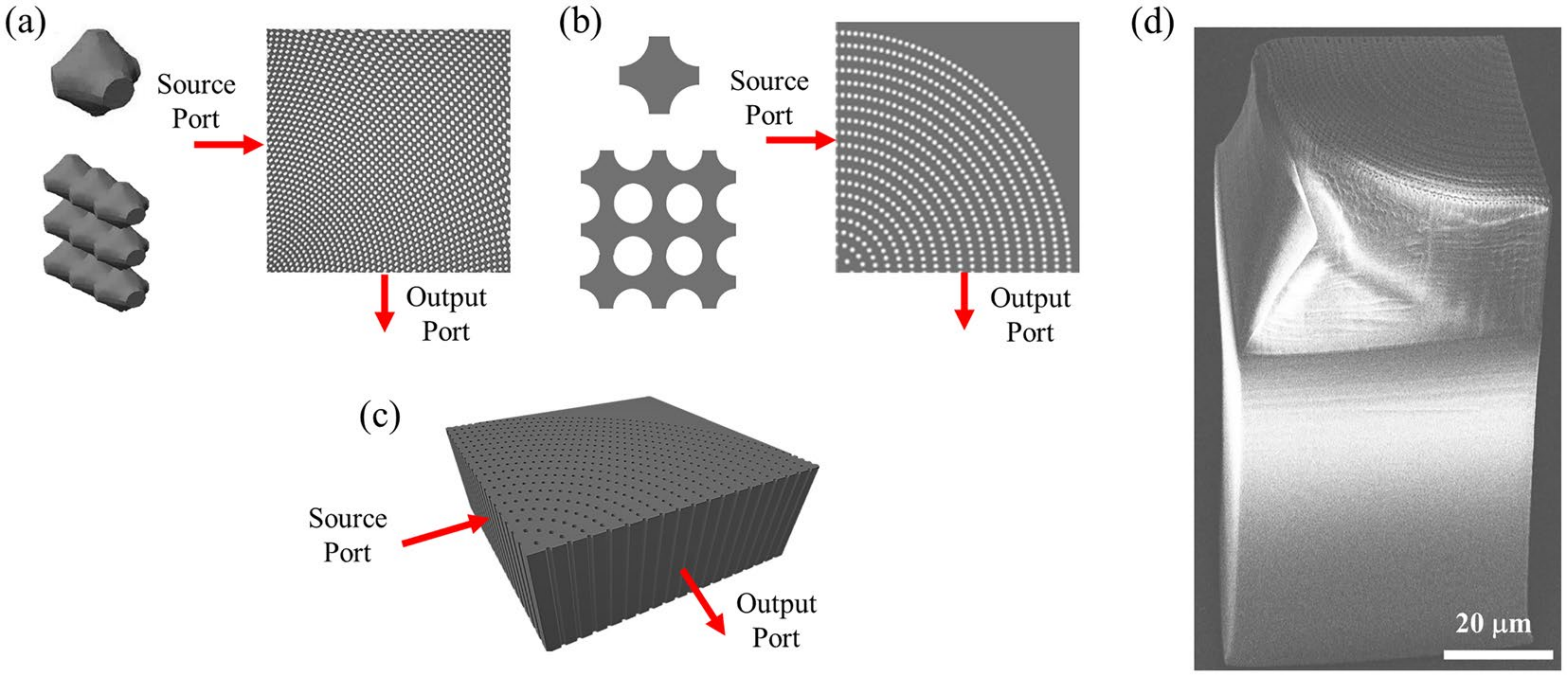
2D and 3D SVPC designs. (**a**) Unit cell, standard lattice pattern, and SVPC for complex structure based on SVLs. (**b**) Simplified 2D unit cell, standard lattice pattern of unit cells, and SVPC for 90-degree bend using polar coordinate function for hole array. (**c**) 3D structures using extrusion of simplified 2D SVPC design for 90-degree bend. (**d**) SEM image of fabricated SVPC prototype atop printing pedestal. Source and output ports are identified for each design.

The initial approach to develop SVPCs follows a similar technique for Spatially Varying Lattices (SVL)^5,17^ presented in prior work^10,14^. At subwavelength scales in the NIR, however, this model is inefficient due to its fragility, resolution requirements, and large computational time for printing and simulation. Our second approach employs a new method using spatial and angular variation through polar coordinates to place a series of holes through a substrate mimicking the SVL unit cells to develop a simplified 2D SVPC design to control light through 90-deg bends and to show how Multiple-Input, Multiple-Output (MIMO) devices could be connected in parallel using a network of these structures. Spatial variation can be implemented to design SVPCs capable of guiding light through sharp bends. Instead of distorting unit cells of the lattice, additional cells are added to maintain spacing throughout the structure. Using a hole diameter of 1 μm and x:y spacing of 1:3 μm, we develop PCs with *ff* = 0.738. Unit cells and accompanying SVPCs for complex and simplified models are shown in Fig. 1a,b, respectively. These figures show the development of the SVPC from individual unit cells, to a small-scale non-varying lattice, and finally to a the full SVPC design. A 3D extrusion, or 2D SVPC with defined thickness, is shown in Fig. 1c. While the entire surface of the SVPC can work as an input port for light, an example position for source ports and corresponding output ports are identified in each of these designs. The initial prototype fabricated atop a pedestal using 3D models developed using Python is shown in Fig. 1d.

### FDTD modeling and simulation

The goal of this work is to demonstrate SVPCs operating in the NIR spectrum (700–1000 nm). To achieve high transmission within this range, low-refractive index materials^1^ with negligible extinction coefficients such as silicon dioxide and photopolymers such as SU-8 or IP-Dip are considered^18–20^. Using low-index materials and subwavelength scales, various unit cell parameters are designed to find optimal designs based on resultant IFCs and bandgaps. We calculate IFCs, bandgaps, and optical properties for SVPCs using FDTD in the open-source software Massachusetts Institute of Technology (MIT) Electromagnetic Equation Propagation (MEEP)^21^ using approximated properties for IP-Dip as the low-index material for each SVPC. This material is commercially available and often used for fabricating 3D nano- and microscale structures. Using simplified structures allow them to be less computationally intensive and more feasible for successful fabrication and implementation in modern optical systems.

**Figure 2:**
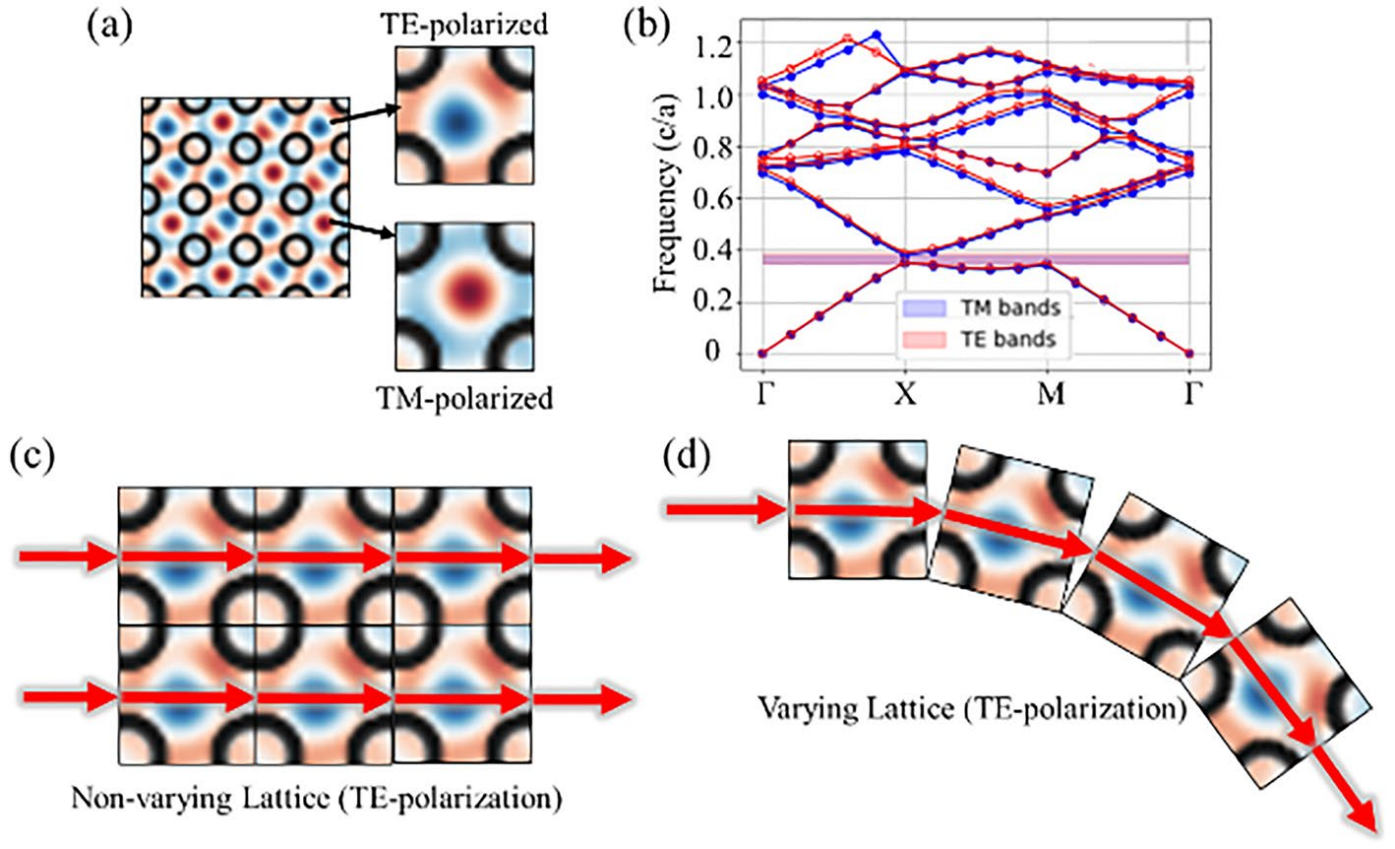
IFC and bandgap simulation results with impact on beam propagation. (**a**) IFCs on non-varying PC lattice for both TE and TM polarization. (**b**) Resulting bandgap of unit cells for non-varying PC lattice. (**c**) Theoretical beam propagation through PC without spatial variation for TE polarization. (**d**) Theoretical beam propagation through PC with spatial variation.

Simulations to analyze IFCs and bandgaps are performed in MEEP using non-varying PC lattices with uniform unit cell structures. Figure 2a, provides the IFC for Transverse-Electric (TE) and Transverse-Magnetic (TM) polarizations of light^22^. Resulting bandgaps for the PC lattice are shown in Fig. 2b, depicting which wavelengths will be transmitted through the structure, and which will be blocked^16^. This plot shows that light within the NIR can be successfully transmitted through the structure and guided by the IFCs of each unit cell. It is important to note that this plot uses a unique set of units and scales based on the MEEP simulations as all units are divided by the speed of light to provide a uniform scale. Figure 2b presents the units of frequency as [*c/a*] where *c* is the speed of light with units of *m/s* and *a* is the unit cell scale with units of μm. This unit can be simplified to a frequency of Hz (1/s) with a scale of 1 × 10^6^, fitting more standard units. Using a unit scale (*a*) of 1 micron, we design our SVPC to operate specifically in the NIR spectrum. Based on this principle, light will propagate through the non-varying PC lattice normal to each IFC and remain collimated as shown in Fig. 2c. Spatial variation can be added to guide light in specific directions as long as the angular distribution does not exceed the limitations of these IFCs. Figure 2d shows how angular distribution for spatial variation maintains collimation through the IFCs of each unit cell while slowly guiding light through sharp bends. These results are directly influenced and controlled by altering the spacing, hole size, or *ff* of the PC structure.

**Figure 3:**
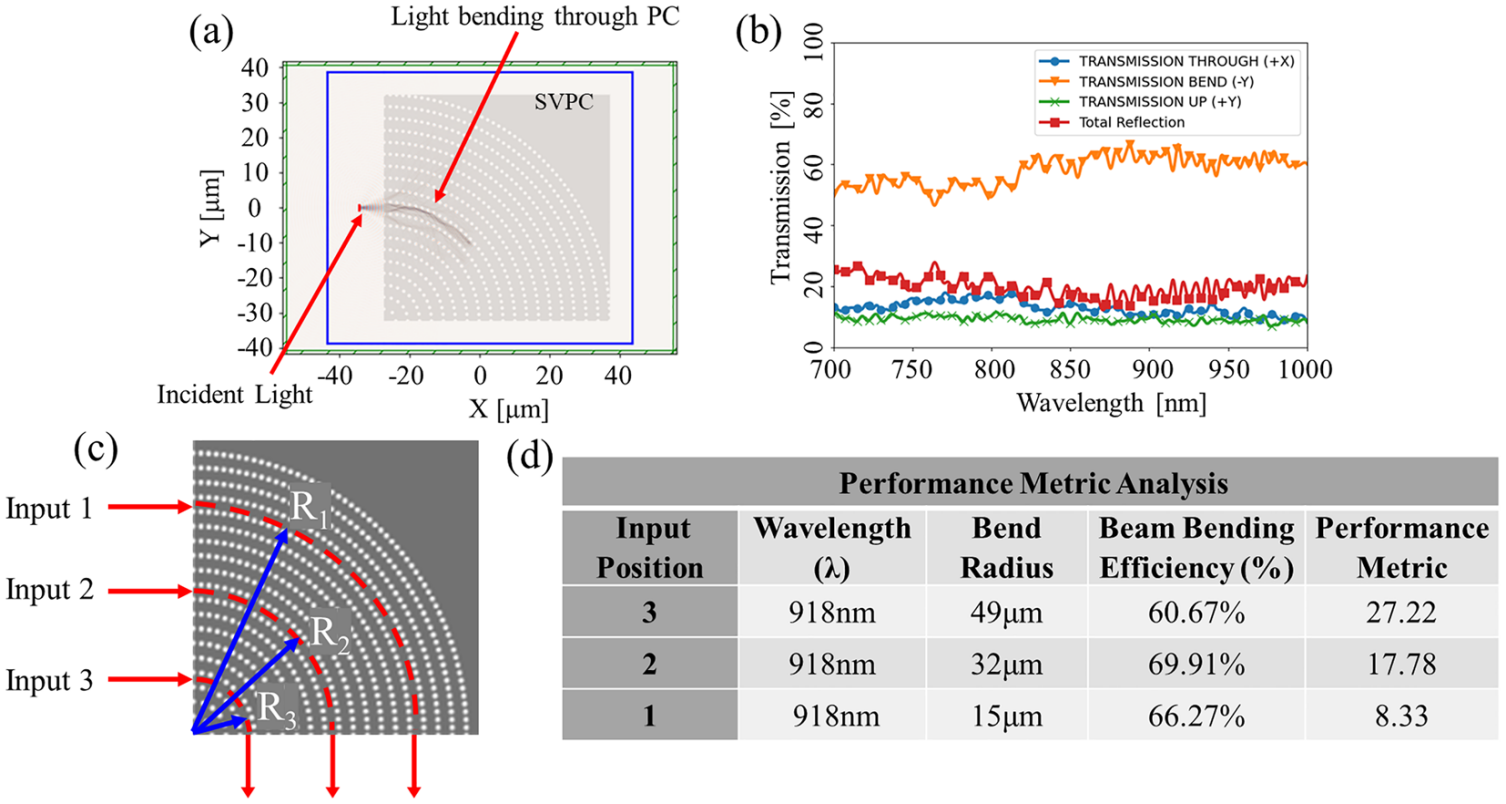
2D simulation results from FDTD simulations in MEEP for 90-deg bend. (**a**) Propagation of light through SVPC for 90-degree bends. (**b**) Transmission properties for SVPC in NIR spectrum. (**c**) Input positions for PM analysis. (**d**) Results of PM analysis and comparison with bending efficiency and parameters.

These simulation results provide a theoretical foundation and validation for SVPCs to guide light through sharp bends with high transmission in the NIR spectrum. Following this validation, we perform simulations on our simplified 2D model for SVPCs shown in Fig. 1b. Figure 3a shows light propagation through the SVPC, while Fig. 3b shows the transmission and reflection properties of the SVPC design. Both Gaussian pulses and Continuous Wave (CW) sources are able to achieve similar results for transmission and reflection data. These simulations are evaluated to determine a Performance Metric (PM) for our SVPCs. The PM used in this work was first suggested by Digaum et al.^6^ to evaluate how effectively an SVPC can direct light through sharp bends. Parameters for this evaluation are shown in Fig. 3c, and the results are given in the table of Fig. 3d.

The 2D SVPC for guiding light through 90-degree bends achieves a beam-bending efficiency between 46–70% throughout the NIR spectrum. Reflection wavers between 12 and 28%, while light directed in other directions—opposite or through the SVPC—ranges from 15 to 30%. A maximum beam-bending efficiency of 69.91% is achieved at 918 nm. The PM analysis shows how one must prioritize either higher efficiency or shorter bends and lower spacing. Input position 1 in Fig. 3c had the best (lowest) PM value of 8.33 while maintaining a relatively high bending efficiency of 66.27%, meaning we can reduce the size or change the input position to improve the PM and implement the system in small-scale devices while maintaining high efficiency. The PM values found for our SVPCs in the NIR spectrum are on the same order of magnitude as those found for similar devices in IR and microwave applications found by Digaum et al.^6^, validating these SVPCs as a viable option for controlling and directing light. Despite high bending efficiencies, the reflection at the input face still has a negative impact on the system. Anti-Reflection Coatings (ARCs) can potentially reduce reflection at the surface of the SVPCs to help increase transmission. Using ARCs in SVPCs for the NIR spectrum will build upon prior work^23,24^ and be reported in the future as we continue to optimize these SVPCs and implement them for specific applications.

These 2D models and simulations demonstrate high transmission properties; however, 3D simulations are required to show how a prototype SVPC may operate in laboratory environments. These simulations are performed using 3D extrusions of the 2D SVPC designs, shown in Fig. 1c. Through 3D simulations in MEEP, our structure, comprised of IP-Dip, maintains self-collimation and directs 31.7% of light through a 90-degree bend at λ = 918 nm. This value shows a disadvantage of the simplified 3D extrusion of the 2D model. Moving from a 2D design to a 3D simulation domain causes the structure’s beam-bending efficiency to decrease by nearly 38%. A lack of spatial variation in the third dimension inhibits the SVPC’s ability to maintain collimation along each axis. Though the results from this 3D approach are lower than the 2D results at this wavelength, the model uses parameters that are viable for fabrication, meaning the experimental results should be similar to these 3D simulation results.

### SVPC fabrication

Our SVPC prototype to control light through 90-degree bends is based on simulations performed using a 3D extrusion of the 2D SVPC designs. The prototype is fabricated with commercially available 3D printers using Two-Photon Polymerization (2PP) with scanning speed and laser power optimized for a proper fabrication recipe. The SVPC prototype is fabricated atop a solid pedestal, elevating it off the substrate and allowing the user to place optical fibers near the faces of the SVPC without touching the substrate^6^. Figure 1d shows a Scanning Electron Microscope (SEM) image of an SVPC prototype consisting of a 32.25 μm tall SVPC with a length and width of 64.5 μm atop a 100 μm tall pedestal. The SVPC prototype used for experimental measurements is identical to that shown in Fig. 1d with an array of 1 μm diameter cylindrical holes, but using a 250 μm tall pedestal, providing additional spacing for fiber alignment.

### Prototype characterization

**Figure 4:**
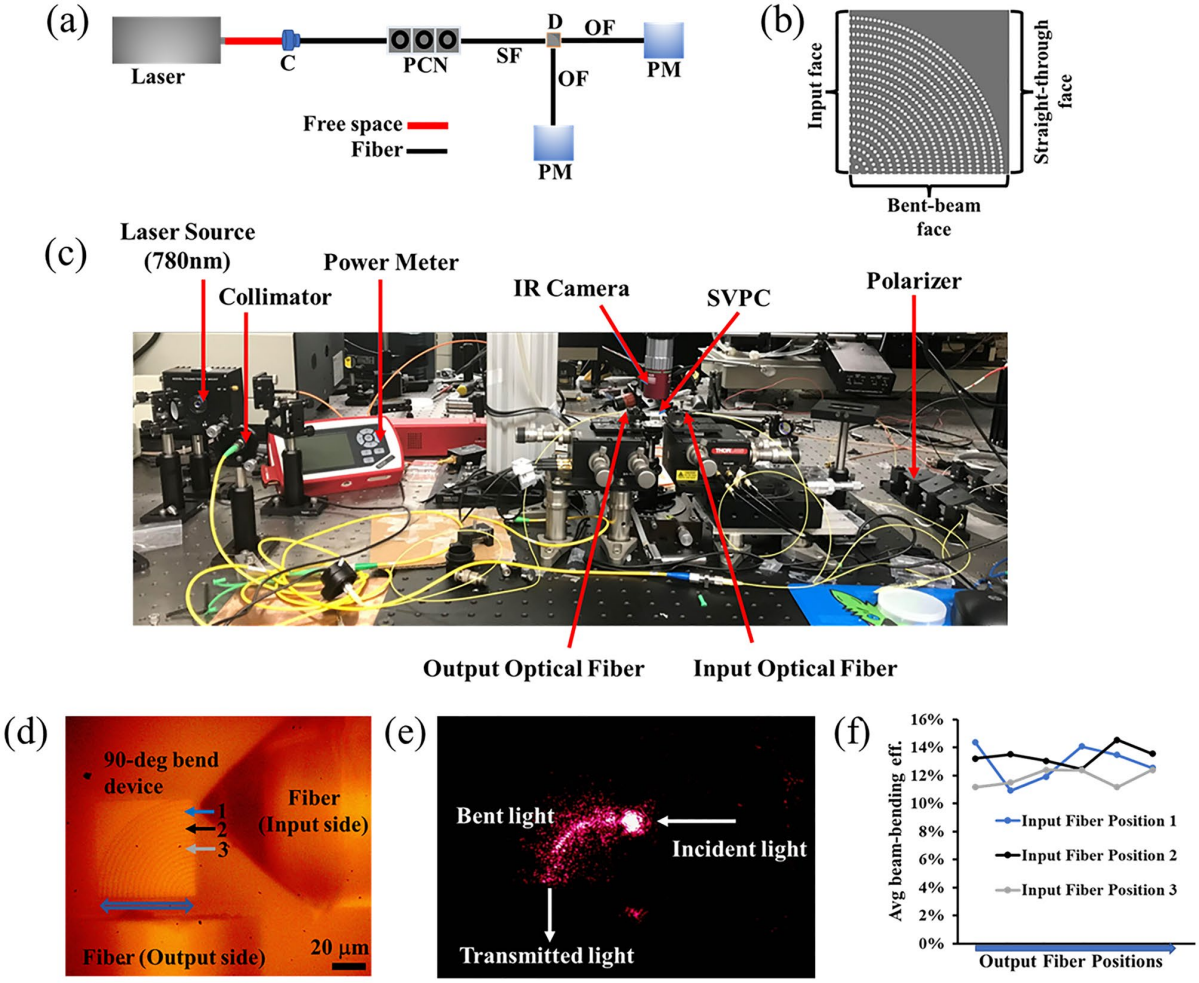
Beam-bending efficiency of the SVPC device. The objects are not drawn to scale. (**a**) Schematic of the experimental setup for characterizing the SVPC device (C: collimator; PCN: polarization controller; SF: source fiber; OF: output fiber; D: SVPC device; and PM: power meter). (**b**) SVPC face identification. (**c**) Experimental setup for prototype SVPC devices. (**d**) Microscope image of the prototype SVPC with the optical fibers for measuring 90-degree beam-bending efficiency. (**e**) Self-collimation and bending of light through the prototype 90-degree bend SVPC. (**f**) Plot of the average beam-bending efficiency; the output fiber is moved from the left to right end on the bent-beam face for measuring the output optical power for each input fiber position.

We use a CW laser source (λ = 780 nm) for characterizing the optical performance of the SVPC prototype using the experimental setup in Fig. 4a. We measure light exiting the bent-beam face as well as the straight-through face using matching multimode output fibers. The multimode fiber with a larger core diameter (50 μm) is used at the output side, ensuring that it collects a majority of light emanating from the output faces. Figure 4b shows each face of the SVPC device. The experimental setup developed to perform this testing is shown in Fig. 4c with all significant components identified. Propagation of light through the prototype SVPC is shown in Fig. 4e. The beam looks dimmer as it travels through the device but becomes brighter at the tip of the output fiber due to surface scattering into the camera; indicating that the device is self-sufficient in bending the laser beam as the surface scattering loss is negligible. To ensure that no damage is incurred by the device or fiber, we keep the fiber tip at a safe distance from the device itself, causing significant diffraction loss before reaching the output fiber tip; implying that our current measurement gives the lower bound of beam-bending efficiency.

The beam-bending efficiency of the prototype SVPC is the ratio of total optical power exiting the bent-beam face to the total optical power incident on the SVPC input face. For each position of the input fiber, the output fiber at the bent-beam face is gradually moved to different positions while measuring optical power. This process is repeated for various input fiber positions as shown in Fig. 4d. Figure 4e demonstrates that light remains self-collimated in the X–Y plane as it propagates through the 90-degree bend. Figure 4f plots the average beam-bending efficiency of the SVPC, reaching as high as 14.4%. The optical power of light exiting the straight-through face is approximately 4 times smaller than that of light emanating from the bent-beam face, indicating that our SVPC strongly directs light through the 90-degree bend.

We can compare these experimental results with simulations using parameters matching our experimental conditions at λ = 780 nm. Using a 2D simulation domain, we achieve a transmission of 52.1%. In a more realistic 3D simulation domain, our SVPC only achieves a beam-bending efficiency of 34.36%. This comparison shows that the experimental results are slightly lower than those obtained from the 3D simulations performed on an extrusion of the 2D SVPC structures, possibly a result of higher insertion loss, scattering, and coupling loss^6^. Scattering, crosstalk, and limitations in efficiency are a result of a combination of factors including adiabacity, fabrication imperfections, and lack of 3D bandgap control. The main limitation of this design is that as an extrusion of a 2D structure, there is no control over propagation in the z-direction, so while light is controlled through the bend in the x and y dimension, spreading in the z dimension reduces the detection by the optical fiber. This was not detected in simulation as the monitor collected 2D data. A comparison between the experimental results and the various simulation models for the 2D and 3D simulations are shown in the Table 1. Our results presented in prior published work using SU-8^10,14^ are provided as well for additional perspective and validation of these results.

**Table 1:** Comparison of beam-bending efficiency of the SVPC Device.

2D SVPC result comparison					
Test	Method	Model Type	Material	Wavelength (nm)	Beam bending efficiency (%)
1	2D simulation	2D	SU-8	780	58.9
2	2D simulation	2D	IP-Dip	780	52.1
3	3D simulation	2D extrusion	IP-Dip	780	34.4
4	Laboratory experiment	Fabricated 2D extrusion	IP-Dip	780	14.4

Table comparing 2D SVPC model types, materials, and bending efficiencies using different simulation and testing methods at 780 nm.

### Prototype testing for potential applications

SVPCs can be applied to connect MIMO devices for optical network applications. Using a network of SVPC devices, a single system could be used to connect a range of optical devices that can be implemented or swapped out at a later time. This has the potential to increase efficiency while decreasing the size of the overall system and enabling it to be customized for specific applications and requirements. In these applications, crosstalk significantly affects the performance of an optical network, often caused by an optical filter or demultiplexer that selects the desired channel but is unable to perfectly reject the neighboring channels. In optical switches, crosstalk occurs due to imperfect isolation between different wavelength ports^25^.

**Figure 5:**
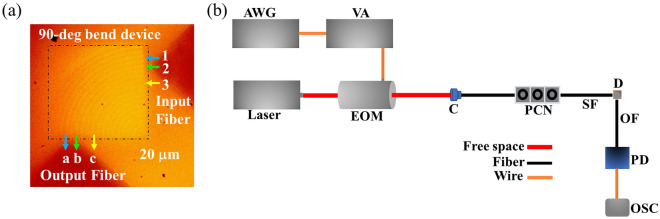
Prototype testing for specific SVPC applications. The objects are not drawn to scale. (**a**) Microscope image of the prototype SVPC with optical fibers for measuring crosstalk. Both fibers are single-mode optical fibers with tapered tips; the numbers and letters indicate the positions of the optical fibers. (**b**) Schematic of experimental setup for demonstrating application of the SVPC with a pulse signal (AWG: Arbitrary Waveform Generator; VA: Voltage Amplifier; EOM: Electro-Optic Modulator; PD: Photodetector; and OSC: Oscilloscope).

We perform measurements to determine the extent of crosstalk within the SVPC as shown in Fig. 5a for 3 input–output channels that could be connected simultaneously using single-mode optical fibers with tapered tips. For each input fiber position, the output fiber is placed where beam collection is maximized between two channels. This crosstalk experiment is novel in its application to PCs. The laser beam is propagated through a specific channel, and the output optical power is measured at different positions. The beam-bending efficiency at each output other than the desired input-to-output channel is calculated to determine the corresponding crosstalk. The crosstalk between positions “a” and “c” is very low (approximately 0.47%) due to the self-collimation and control of light by the SVPC. This is repeated for each position, and the corresponding crosstalks are shown in Table 2a. The minimum value obtained from our measurements was approximately 0.34%, indicating that the prototype SVPC has good resistance to crosstalk. We further show the isolation of each channel where a maximum is found for channel 2-b. This analysis of crosstalk and channel isolation can be used to set boundaries and tolerances for independent channels. While in theory, an ideal IFC can set each channel, or layer in the SVPC, to be completely independent, we see there is some slight leaking. This initial testing has shown minimal crosstalk between three specific channels; however, this work could be expanded to narrow down and identify the true width of independent propagation. For example, if we define less than 1% as a completely independent channel, then we can identify the bounds of each channel to maximize the amount of simultaneous data transfer possible within a given structure. The PM analysis for various positions of the input and output optical fibers at λ = 780 nm is shown in Table 2b. The best, or lowest, PM value (9.27) is obtained for the input–output channel of position 3-position “c”.

**Table 2:** Experimental results.

(a) Crosstalk and isolation (%)				
Input position	Output position (a)	Output Position (b)	Output position (c)	Channel isolation
1	–	0.69	0.47	98.85
2	0.34	–	0.57	99.09
3	0.55	0.50	–	98.97

(b) Performance metric analysis				
Input–output channel	Bend radius (µm)	PM		
1-a	46.78	23.03		
2-b	37.46	18.44		
3-c	18.83	9.27		

**a** Crosstalk measurement data for various positions of the input and output fibers with resultant channel isolation.

**b** PM analysis for each input–output channel.

We demonstrate one of the potential applications of SVPCs by sending a pulse signal and comparing it with the signal obtained at the bent-beam face in terms of the Signal-to-Noise Ratio (SNR). The SNR helps determine the capability of a channel to transmit information in standard optical networks. External modulators, such as Electro-Optic Modulators (EOMs) are used to impose the information signal to be transmitted. Here, an information signal is used to modulate a CW signal generated by a NIR laser source before it is transmitted over the optical fiber^25^. For this purpose, we used the experimental setup shown in Fig. 5b.

For optical communication systems, a high SNR and low Bit-Error-Rate (BER) are preferred for better reliability. The measured SNR at the input and output faces of the SVPC prototype are 16.68 dB and 11.32 dB, respectively, indicating that the SVPC is capable of sending pulse signals with a low-BER. The SNR at the output side is lower than that of the input side. The SNR values are not as high as a typical optical communication system (~ 30 to 40 dB) due to noise from the EOM and photodetector which can be improved in future work. This experiment is a preliminary demonstration of how effectively a pulse signal can be transmitted through an SVPC. Once we demonstrate the possibility to connect input–output channels reliably, actual modulated data can be sent between different channels.

### Future applications of SVPCs in the NIR

**Figure 6:**
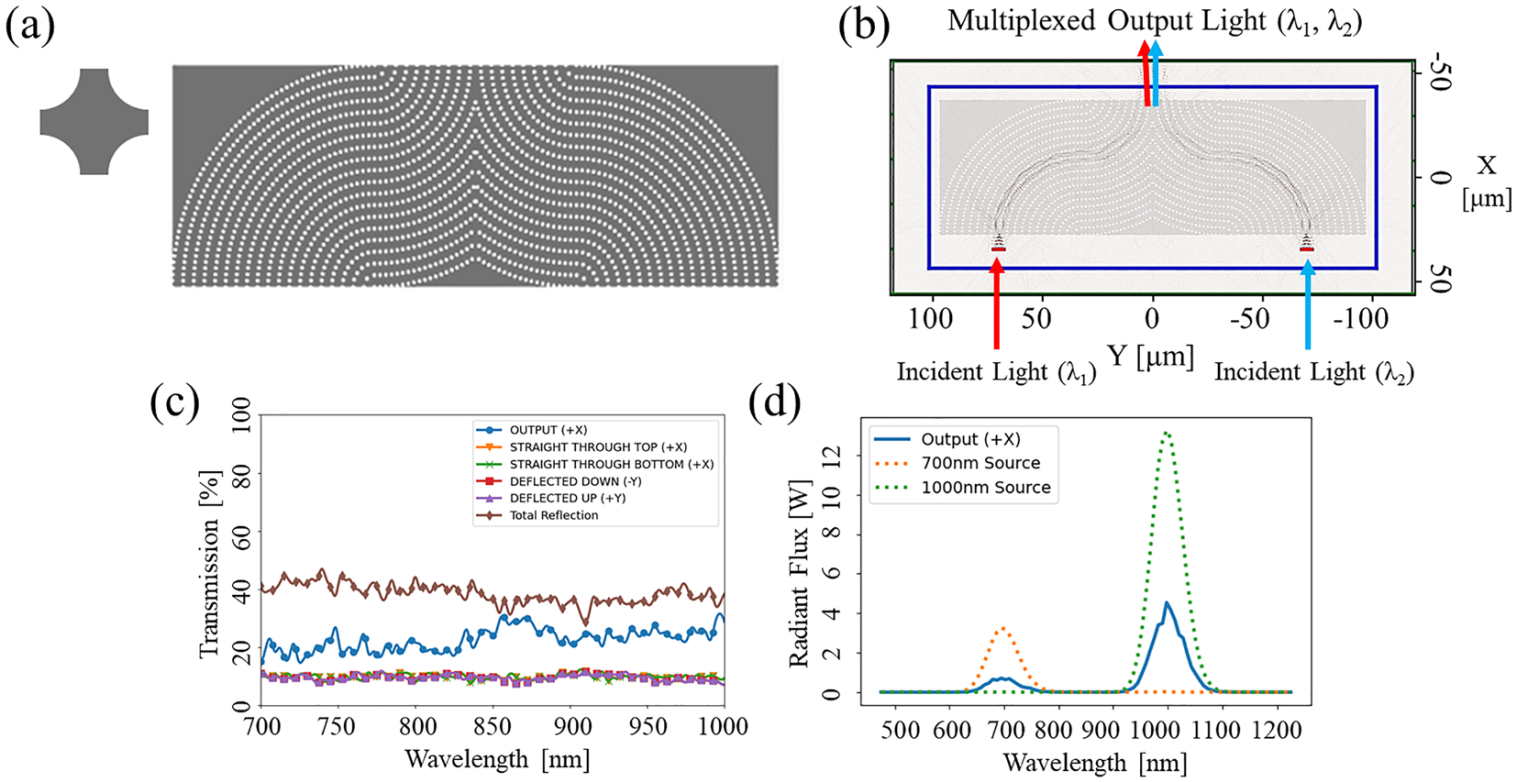
2D simulation results from FDTD simulations in MEEP for OR logic gate. (**A**) Unit cell and full OR logic gate using SVPC design with spatial variation following 90-degree bend. (**B**) Propagation of light through SVPC for OR logic gates. (**C**) Transmission properties for SVPC in NIR spectrum. (**D**) Energy of two distinct wavelengths (λ_1_ = 700 nm and λ_2_ = 1000 nm) from multiplexed output beam (solid line) compared to input beam (dashed lines).

SVPCs have potential applications for structures such as OR logic gates and frequency-selective surfaces. As multiplexing devices, SVPC-based OR logic gates are capable of combining multiple similar or dissimilar wavelengths into a single output^13,26^. This operation has previously been shown in 1D and 2D devices^27^. These structures can be made using a similar variation of unit cells as the SVPC designed for controlling light through 90-degree bends. Figure 6a shows the unit cell and full OR logic gate developed using this approach. Figure 6b shows the light propagation through the SVPC using IP-Dip, and Fig. 6c shows the transmission and reflection properties of the SVPC design. Figure 6d depicts the SVPC operating as an OR logic gate to multiplex output light, showing two distinct wavelengths at the output of the model.

Our device shows the ability to combine the amplitudes of two different light sources. The SVPC for OR logic gates operating as a multiplexer, achieves between 15 and 32% transmission through the structure across the NIR spectrum. The symmetry of this SVPC design causes the amount of light deflected opposite of the bend as well as straight through the structure on the top and bottom interfaces to be the same, meaning their plots overlap around 10% for both faces. The largest portion of light throughout a majority of the NIR spectrum is reflected at the input, accounting for 28–47% of total transmission. The maximum transmission value of 31.55% is achieved at 997 nm for the OR logic gate. This capability can be improved through limiting crosstalk between different incident positions throughout the SVPC and implementing ARCs at the input face of the SVPC. The device currently experiences ~ 50% loss and would require a thresholding device at the output to achieve desired operation for optical OR logic gates. Continued work will expand this structure to control light in all three dimensions to improve the transmission of the system by preventing scattering in the z-dimension as well as open the potential for additional input channels to transmit and multiplex additional sources of light which is not possible in standard 1D and 2D devices.

## Discussion

In this work, we demonstrate that specially designed SVPCs can achieve unique IFCs and bandgaps to control self-collimation and direct light through sharp bends in the NIR spectrum through rigorous research, modeling, simulation, fabrication, and testing. While basic optical properties such as transmission and reflection have shown basic control over light propagation, new testing methods have been developed to show potential, application-specific benefits of SVPCs. Comparisons between the beam-bending efficiency and PM of different SVPCs and input positions through both simulations and experiments are used to evaluate the benefits and drawbacks of SVPCs in certain scenarios. Experiments showing limited crosstalk and improved pulsed signal transmission with SVPC designs demonstrate their potential for implementation into next-generation optical systems such as multiplexers, logic gates, and optical switches. The testing methods and accompanying results provide a novel, beneficial method for predicting the unique functionality of simplified 2D SVPCs.

The capabilities for unique control of light propagation through spatial variation and self-collimation are relatively unexplored for SVPCs in the NIR spectrum. One application of such capabilities is in creating connections between disparate points in a photonic structure without needing a dedicated point-to-point waveguide. Through both simulation and experiment, we were able to show that the device is polarization-independent as switching from TE to TM did not impact the resultant transmission. This was done by changing the orientation of the electric field and simulation as well as adjusting/rotating the polarization rotator in the experimental setup. Adiabatically tailoring holes throughout the SVPC structure allows an unguided beam to flow through the 90-deg bend with small radii. Using multiple channels through the SVPC, can connect different inputs and outputs by utilizing the self-collimation and beam control properties of the SVPC without a waveguide, which is a novel approach in the NIR spectrum. This control of light through multiple channels in a single structure and single fabrication run can significantly reduce the size of optical devices and the complexity of fabrication, enabling the development of dual-use devices and provides potential improvements for multiplexing devices and logic gates as shown by our initial results in Fig. 6a–d. An array of these SVPC structures could be assembled to efficiently control light propagating through a complete optical system where specific devices and components can be added or switched out at a later time for unique and variable applications. The ability to link multiple optical components allows these SVPC networks to improve the manipulation and efficiency achieved in optical systems for a range of applications. Future work will demonstrate prototypes for these multiplexing devices and logic gates, showing the new benefits of SVPCs for optical systems in the NIR.

This work shows great potential for real-world applications of SVPCs with self-collimation and direction of light through sharp bends on small scales, however, the lack of spatial variation in the third dimension diminishes the self-collimation and transmission properties for larger structures exceeding the Rayleigh range. We are developing improved structures to control the propagation of light in the third dimension without drastically increasing the complexity of the model so that it can still be easily fabricated using the 2PP technique. We will follow a similar process for modeling, simulating, fabricating, and testing the improved 3D models to show the full potential of enhanced optical systems using 3D SVPCs. We will investigate the characterization of optical properties, crosstalk, and specific applications such as improved optical communication or ranging. This future work will demonstrate methods for more precise control of light in SVPCs for directing light in the NIR spectrum.

## Methods

### SVPC modeling

The modeling approach builds upon prior work^10,14^; however, the structure is simplified to make it more feasible for fabrication and implementation in specific devices. Instead of stretching or distorting each unit cell and corresponding holes, additional cells are added based on the layer distance in order to maintain the same spacing of holes and unit cells, preserving the bandgap and self-collimation properties of the PC as spatial variation is introduced. The *ff* for these SVPC structures is based on the spacing and size of the unit cells defined for the PC and is maintained during spatial variation. This *ff* is a ratio of the PC structure once holes are removed to the full region of the device. While we choose to set the spacing and size of holes to then determine the *ff*, users could also set the *ff* to determine values for spacing or the size of holes in their own PCs. The *ff* is taken for the region of operation, meaning the large substrate region in the upper-right portion of Fig. 1b is not taken into account. The optical properties such as the effective index and direction of light through the SVPC is directly impacted by *ff*. Geometries for the simplified 2D and 3D models are built using MEEP’s geometries, such as circles, rectangles, and cylinders, to mimic unit cell structures by arranging them based on equations we have developed. The simplicity of these designs allows them to be less computationally intensive and more feasible for successful fabrication through the 2PP technique and Nanoscribe 3D printers.

### FDTD simulation approach

2D and 3D simulations are performed in the open-source FDTD simulation software MEEP. As open-source software, MEEP allows a wider range of users to test unique structures without the high cost of standard, commercial software. Initially, constant material parameters are set to match those of the Nanoscribe 3D printer’s fabrication material, IP-Dip. Using constant material parameters reduces the resolution requirements in order for simulations to run more efficiently. This assumption is also a fair estimate as the material’s properties are relatively constant with a low refractive index and a negligible extinction coefficient. STereoLithography (STL) files are created matching the models in the 2D and 3D simulations and used to fabricate prototypes for testing. The simulations use input parameters based on the experimental setup: centered position, 0° (normal) incidence angle, beam width of 6 μm, and λ = 780 nm. Flux monitors are added at each face of the SVPC structure to find the transmission through each face as well as the reflection directions. The total propagation through each monitor is added together in order to find the relative percent transmission and reflection through each surface of the SVPC. We can calculate the beam-bending efficiency by dividing the amount of energy propagating through the 90-degree bend by the total amount of energy emitted by the source and present in the simulation domain. Additional monitors are added to the 3D simulations to calculate the propagation in the extra dimension. The blue and green lines in Figs. 3a and 6b show the z-component of the electric field as light propagates through the SVPC. Both Gaussian pulses and CW sources are able to achieve similar results for transmission and reflection data. Simulations are set with long runtimes or until the source fields have decayed in order to allow for significant simulation time for accurate flux data through all monitors.

### Performance metric

In both simulations and experiments, we look at the PM for the SVPCs^6^. This PM value takes into account the required bend radius to direct light for a specific wavelength and refractive index contrast. The following equation is used in this work to calculate the metric:

$$PM=\frac{{R}_{bend}}{{\lambda }_{0}}(\Delta n)$$

where ${R}_{bend}$ is the bend radius used in the model, ${\lambda }_{0}$ is the wavelength of interest, and $\Delta n$ is the refractive index contrast. The refractive index contrast is found by comparing the effective index of the PC with that of air. Since our structure uses holes of air, the effective index of the PC can be approximated to have a linear relationship to the *ff*^28,29^. Using this principle, we can use the calculated *ff* to determine the refractive index contrast for the model^30^:

$$\Delta n={n}_{PC}-{n}_{air};{ n}_{PC}={n}_{air}+ff({n}_{substrate}-{n}_{air})$$

$$\Delta n=ff\left({n}_{substrate}-{n}_{air}\right).$$

As the PC is constructed from IP-Dip with air holes using *ff* = 0.738, the refractive index contrast for the model is 0.384. Using this contrast and the PM equation, the PM can be calculated and compared for different conditions and bend radii to see which model or input position is the best for a given scenario. For example, one designer may choose smaller size requirements and is willing to sacrifice some bending efficiency in order to improve the PM, while another user may not have the same size requirements and will sacrifice the PM to improve the beam-bending efficiency. The PM values found for our SVPCs in the NIR spectrum are on the same order of magnitude as those found for similar devices in IR and microwave applications found by Digaum et al.^6^, validating these SVPCs as a viable option for controlling and directing light.

### Fabrication technique

The SVPC prototype is fabricated on a glass substrate with a negative tone photoresist (IP-Dip, Nanoscribe GmbH & Co. KG) using a commercially available 3D laser writing lithography system equipped with a femtosecond laser at λ = 780 nm (Photonic Professional GT, Nanoscribe GmbH & Co. KG). IP-Dip is a photoresist specially designed for Nanoscribe’s Dip-in Laser Lithography (DiLL) technology^31^. This technique is capable, in principle, of ~ 300 nm lateral resolution and ~ 500 nm vertical resolution. The objective of the microscope is immersed directly into the liquid photoresist. IP-Dip acts as a photosensitive material and also as an immersion medium, providing ideal focusing and a high resolution (down to approximately 200 nm) because of its refractive index matching with the focusing optics^32^. After the writing step, the sample is immersed in a bath of a developer (Propylene Glycol Monomethyl Ether Acetate, Sigma-Aldrich) and subsequently rinsed with isopropyl alcohol to remove the unpolymerized photoresist; it is then allowed to dry in air. The features of the prototype SVPC are inspected from a SEM image to assess and compare their structural forms to the designed structures.

### Experimental procedure

The experimental setup for studying the optical performance of the SVPC prototype is shown in Fig. 4a. The glass substrate that has the fabricated SVPC prototype is mounted onto a sample stage. The laser beam is steered by the mirrors into a beam collimator which is then passed through a polarization controller and coupled with the source fiber. The light emanating from the device is collected by the multimode output fibers. A single-mode optical fiber with a tapered tip (THORLABS) is used as a source fiber to launch the laser at the input face of the device. A combination of an objective lens and a camera is used to view and align the device and the optical fibers. Each optical fiber is mounted on a three-axis translation stage (THORLABS, NanoMax 300) that allows it to position and move the fiber precisely to the proximity of the SVPC prototype’s face. The power of the laser beam incident into the device and those collected by each output fibers are measured using an optical power meter (THORLABS, PM100D).

### Crosstalk evaluation

Single-mode optical fibers with tapered tips (OZ Optics TSMJ-3A) are used for the crosstalk measurements as shown in Fig. 5a. The numbers 1, 2, and 3 indicate the positions of the optical fibers at the input side, while the letters “a,” “b,” and “c” represent positions of the optical fibers at the output sides corresponding to each input. As an example, when the input fiber is at position 1, the output fiber is moved to a position where the optical power is maximum, in this case, position “a,” meaning two new channels are connected. The output optical power at positions “c” is also measured, while the input is at position 1. These ratios are calculated to determine the crosstalk. Additional calculations were performed to define the isolation achieved by each defined channel.

### Demonstration with pulse signals

Figure 5d shows the experimental setup used for demonstrating the applications of the SVPC for sending a pulse signal. A CW laser (λ = 820 nm) is sent to a free-space EOM. The output from an arbitrary waveform generator is connected to a voltage amplifier that amplifies the voltage of the signal from the waveform generator and provides a bias voltage to the EOM. The EOM modulates the laser beam according to the signal’s nature from the waveform generator to encode real data on the optical signal. The laser beam from the EOM is steered by the mirrors into a beam collimator which is then passed through a polarization controller and coupled with the source fiber; the beam is collected by an output fiber from the bent-beam face. The beams at the input and output are detected using a photodetector (THORLABS, D400FC) and recorded by an oscilloscope, then finally compared to the input signal.

## Data Availability

Data underlying the results presented in this paper may be obtained from the authors upon reasonable request. Please contact Dr. Rudra Gnawali to request data or information relating to this study. His contact information is provided in the author section at the beginning of this document.
